# Antimicrobial genes from *Allium sativum* and *Pinellia ternata* revealed by a *Bacillus subtilis* expression system

**DOI:** 10.1038/s41598-018-32852-x

**Published:** 2018-09-28

**Authors:** Xi Kong, Mei Yang, Hafiz Muhammad Khalid Abbas, Jia Wu, Mengge Li, Wubei Dong

**Affiliations:** Department of Plant Pathology, College of Plant Science and Technology and the Key Lab of Crop Disease Monitoring & Safety Control in Hubei Province, Huazhong Agricultural University, Wuhan, Hubei Province 430070 China

## Abstract

Antimicrobial genes are found in all classes of life. To efficiently isolate these genes, we used *Bacillus subtilis* and *Escherichia coli* as target indicator bacteria and transformed them with cDNA libraries. Among thousands of expressed proteins, candidate proteins played antimicrobial roles from the inside of the indicator bacteria (internal effect), contributing to the sensitivity (much more sensitivity than the external effect from antimicrobial proteins working from outside of the cells) and the high throughput ability of screening. We found that *B. subtilis* is more efficient and reliable than *E. coli*. Using the *B. subtilis* expression system, we identified 19 novel, broad-spectrum antimicrobial genes. Proteins expressed by these genes were extracted and tested, exhibiting strong external antibacterial, antifungal and nematicidal activities. Furthermore, these newly isolated proteins could control plant diseases. Application of these proteins secreted by engineered *B. subtilis* in soil could inhibit the growth of pathogenic bacteria. These proteins are thermally stable and suitable for clinical medicine, as they exhibited no haemolytic activity. Based on our findings, we speculated that plant, animal and human pathogenic bacteria, fungi or even cancer cells might be taken as the indicator target cells for screening specific resistance genes.

## Introduction

The emerging resistance of pathogenic microbes against antibiotics or their hosts has created a basic need for the development of novel and unique classes of antimicrobial compounds and antimicrobial genes^1,2^. Humans have benefited tremendously from antibiotics; however, the world now has to dedicate itself to solving their consequences, i.e., drug resistance and harmful effects on human health and environmental safety^3^. A number of studies have been conducted and antimicrobial peptides (AMPs) have been proposed as an ideal alternative to traditional antibiotics to solve the emerging issue of antibiotic resistance^4^. Multiple-resistant ‘superbug’ bacteria have the ability to change targets and reduce permeability against antibiotics by using the phenomena of mutations^5^, whereas antimicrobial peptides often do not have specific targets and are capable of disrupting the cell membranes, which gives them durable effects on pathogens and makes them be considered as ideal candidates for clinical exploitations^6,7^. Antimicrobial peptides are available not only against human pathogens but also as promising alternatives for the prevention of plant diseases. Plant resistance can be improved by spraying antimicrobial peptides on the surface of plants or by creating transgenes^8^. As a defense response against the invasion of pathogens, plants upregulate a series of genes as part of their innate immune response^9^. Among them, some genes exhibit a broad range of antimicrobial activities^10^. In addition to affecting the microbial community directly, some genes can regulate the innate immune responses of the host and play indirect roles against pathogens^11^. Antimicrobial peptides encoded by these pathogen infection upregulated host genes are relatively small (<60 amino acids) and have the following advantages: broad antimicrobial spectrum, fast killing, and potential to be used alone or in combination^12^. These unique features have encouraged researchers to establish methods for the discovery of new classes of AMPs and their mimics from different organisms over the last two decades^1,8,13^.

Many screening methods focused on previously known antimicrobial genes^14^, such as those based on homologous sequences^15^, developing genetic algorithms based on existing databases^16^, purifying proteins from biological extracts and then testing for antibacterial activities^17^, or relying on only a few plants whose genomics information is available^18^. These screening methods are generally characterized by a low possibility to screen new types of antimicrobial genes, high cost and being time consuming. Therefore, it is important to construct a strategy to screen antimicrobial genes with high screening efficiency and low cost. Whether on a laboratory or industrial scale for protein production and traditional fermented food, prokaryotic systems are the most widely used systems^19^. One of the advantages of these systems is the ability to obtain large numbers of proteins in a minimum time span. Among them, the *E. coli* expression system is the most commonly used system because of its simple and cheap cell culture, fast growth rate, high transformation efficiency and relatively thorough and clear mechanisms of transcription and translation^20,21^. The *B. subtilis* system is increasingly used because it is considered a safe organism and can directly secrete extracellular proteins into the culture medium^22,23^.

Plants have candidate antimicrobial genes with specific roles in host-pathogen interactions^24^. Garlic, or *Allium sativum*, belonging to the family *Alliaceae*, is a monocotyledonous plant that is considered a safe food source from leaf to root^25^. *Pinellia ternata* (Thunb.) Breit, belonging to the *Araceae* family, is a traditional Chinese medicinal herb reported to treat several diseases including phlegm, cough and vomiting^26^. These plants have been extensively used for many years as culinary agents and as an important ingredient in traditional medicines^27,28^. However, most studies have focused on the antimicrobial and clinical effects of *A. sativum* and *P. ternata* extracts or compounds^29–31^, while ignoring their potential functional antimicrobial genes or proteins. Keeping the previous studies in view, it is necessary to explore *A. sativum* and *P. ternata* genes for antimicrobial potential.

In our study, we have used *B. subtilis* and *E. coli* expression systems to develop an efficient strategy for the functional isolation of novel antimicrobial genes. This strategy is sufficiently efficient to explore the prokaryotic and eukaryotic organisms for the isolation of antimicrobial genes, not only against ‘superbug’ bacteria but also against plant and human pathogens. For plant, animal and human pathogens or cancer cells, if gene transformation systems were developed and it was possible to transform gene libraries into these pathogens or cancer cells, then it would be feasible to screen specific antimicrobial genes against these pathogens or cancer cells.

## Results

### The *B. subtilis* and *E. coli* expression systems were feasible tools for the isolation of antimicrobial genes

In an effort to identify a simple and fast system to screen antimicrobial genes, we developed a strategy that is different from traditional approaches and can efficiently isolate and purify antimicrobial proteins from almost all kinds of organisms. cDNA libraries were constructed using two different expression vectors, pBE-S and pET22-(b), which were transformed into *B. subtilis* and *E. coli* expression systems, respectively. Libraries were evaluated by considering primary library titer, recombination rate and average length of inserts^32^. The primary library titer and recombination rate were 4.6 × 10^6^ pfu/ml and 96.7%, respectively, for the *E. coli* cDNA library and 7.8 × 10^6^ pfu/ml and 91.7%, respectively, for the *B. subtilis* cDNA library (Fig. S1). Quality analysis of both cDNA libraries indicated that the *E. coli* expression library was slightly better than the *B. subtilis* expression library. Altogether, both libraries were successfully constructed and proven to be sufficiently high quality for further experiments.

**Figure 1:**
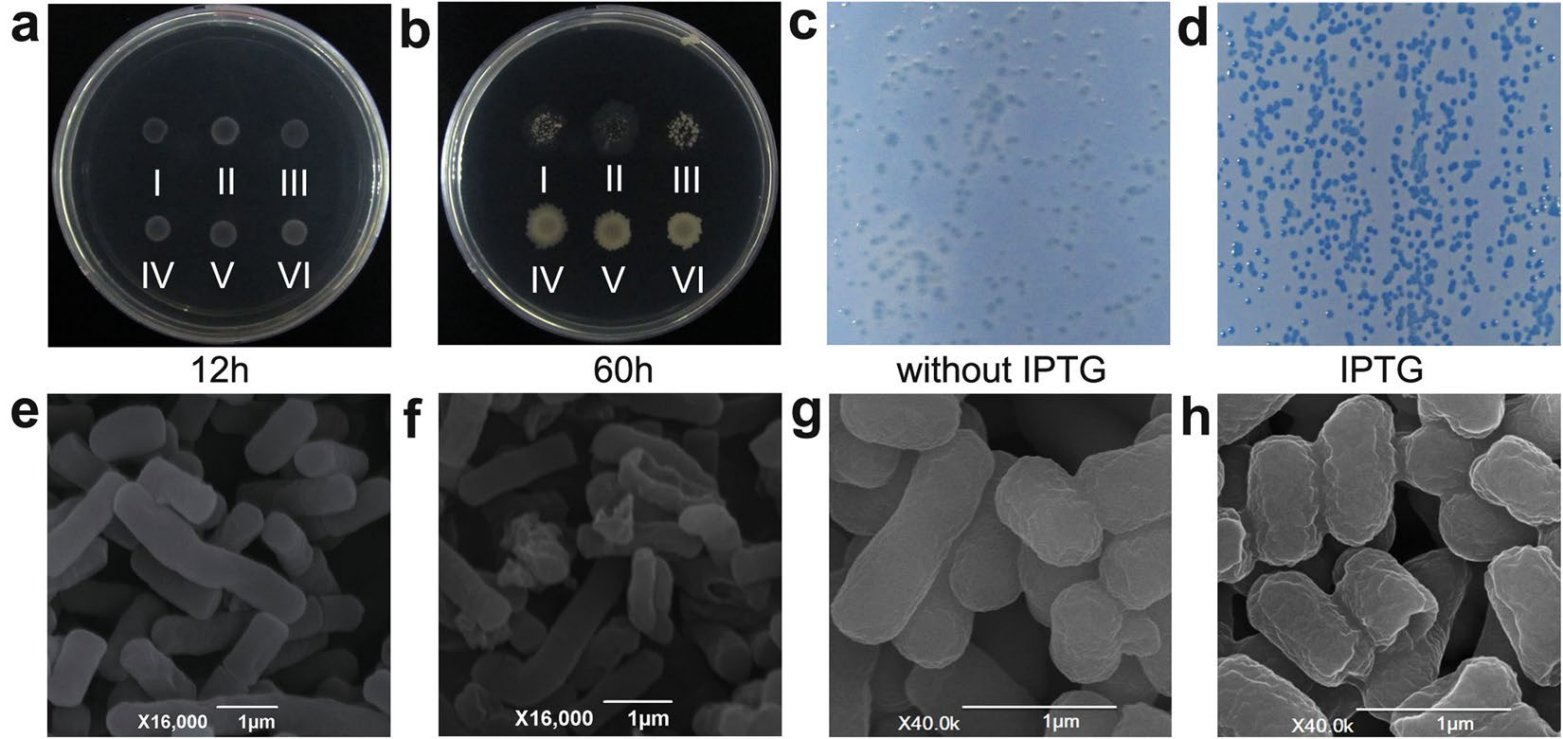
Killing effects of intracellular expression of antimicrobial genes on host cells. (**a,b**) The *B. subtilis* expression system showing autolysis from 12 to 60 h (a and b are the same culture dish). A drop of 2 µl of *B. subtilis* strains was placed on LB plates. Kanamycin was used as screening antibiotic. All colonies **(a**: I–VI) grew normally in the first 12 h, while after 48 h three different colonies **(b**: I–III) with intracellular expression of *AsR*36, *AsR*117 and *AsR*416 genes showed autolysis and three different colonies (IV–VI) showed no autolysis (control). (**c,d**) The *E. coli* strain harboring *AsR*E67 gene stained by trypan blue and bromophenol blue. The strain was spread on LB plates covered with membrane filters. Damaged host cells were stained blue (**d**: cell death was caused by IPTG-induced *AsR*E67 gene expression), and intact host cells were not able to be stained (**c**: control, without IPTG induction and cell death) after growth in the presence or absence of IPTG. (**e,f**) Scanning electron microscopy of *B. subtilis*, (**e**) control colonies no autolysis, and (**f**) autolyzed colonies (AsR416 protein intracellularly disrupted the host cells). (**g,h**) Scanning electron microscopy of *E. coli*, (**g**) unstained colonies as control, and stained colonies (**h**) with AsRE76 caused shrinking cells. Experiments were repeated three times under the same conditions, and similar results were observed.

The starting point for both strategies was based on the fact that *B. subtilis* or *E. coli* host cells will show death or damage if protein products encoded by cDNA libraries are toxic or play antimicrobial roles in cells. Therefore, the screening index was whether or not the host cells were damaged. Trypan blue dye, the most commonly used vital stain, was used in *E. coli* to distinguish viable cells from cells with damaged membranes or dead cells. For the *B. subtilis* cDNA library, a total of 1700 colonies were screened and 48 repeatedly showed autolysis (Fig. 1a,b), while for the *E. coli* cDNA library, 170 positive clones were selected from a total of 2000 cDNA clones after trypan blue staining procedure. Later, 70 clones were found to repeatedly show positive staining (Fig. 1c,d). *AsR*416 and *AsR*E67 were selected from *B. subtilis* and *E. coli* libraries, respectively, and observed by scanning electron microscopy after being cultured for 36 h (Fig. 1e–h). Autolyzed cells caused by intracellular expression of the *AsR*416 gene showed abnormal morphology with significant cell structure change and severe cell damage; some cells were even fragmented (Fig. 1f). IPTG-induced *AsR*E67 *E. coli* cells had a significant change in shape compared to the control, with severe shrinkage and smaller size (Fig. 1h). These results showed that the candidate antimicrobial genes played a significant role in the destruction of bacterial cells.

By using these two screening systems, 118 potential antimicrobial genes were obtained that showed positive antimicrobial activities during all repeated experiments. From these results, it was proved that both screening systems were feasible to use for antimicrobial gene or antimicrobial peptide screening.

### The *B. subtilis* expression system was more effective than the *E. coli* expression system for antimicrobial gene screening

**Figure 2:**
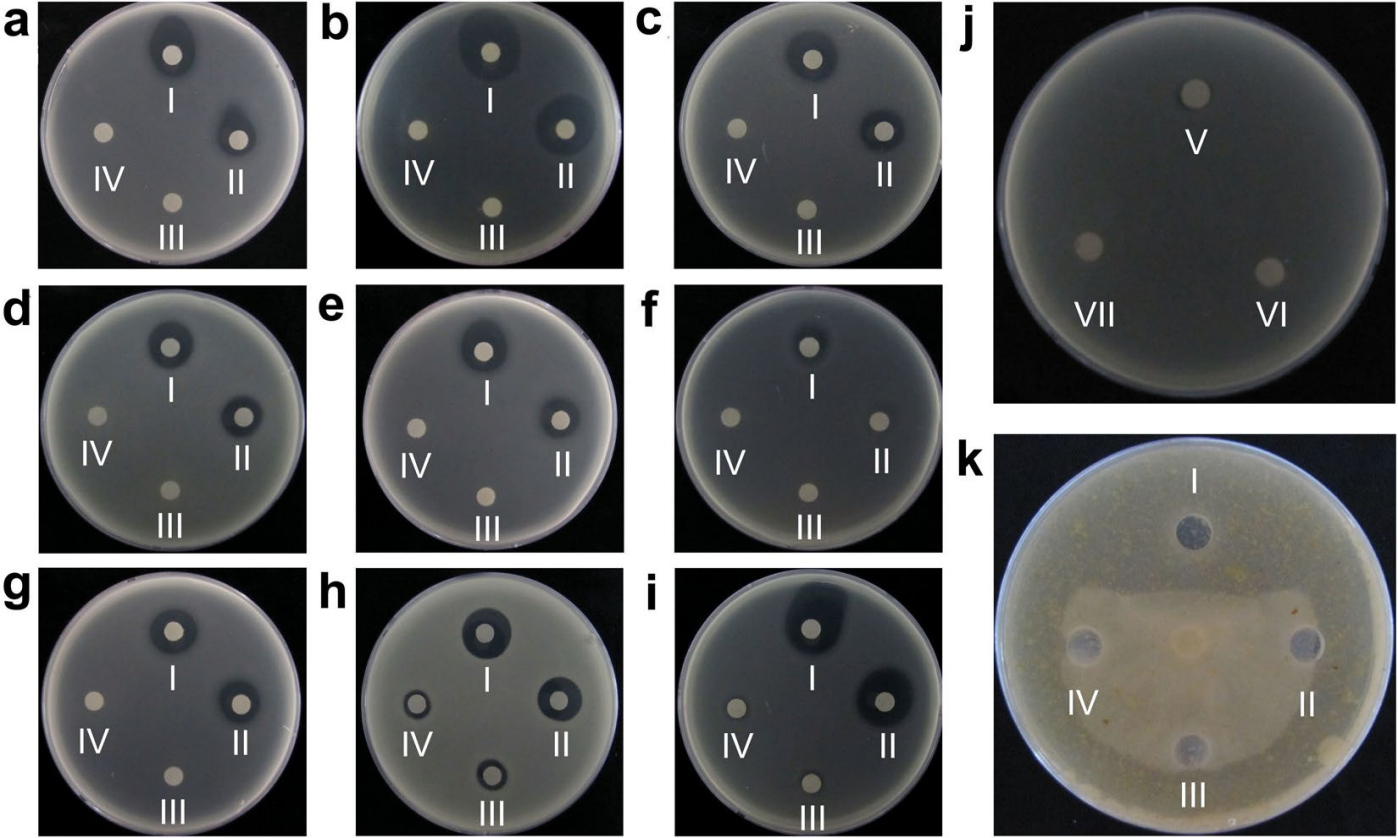
Antimicrobial activities of screened proteins. (**a–i**) Antibacterial effects of proteins screened by the *B. subtilis* system, in which 20 mg proteins extracted by ammonium sulfate were dropped on filter papers, and inhibition zones appeared after 6–12 h. Indicator bacteria are *B. subtilis* 168 (**a**), *B. anthraci* (**b**), *B. cereus* (**c**), *R. solanacearum* (**d**), *C. fangii* (**e**), *B. subtilis* WB800 (**f**), *C. michiganensis* subsp. *michiganense* (**g**), *B. subtilis* 330-2 (**h**), and *C. michiganensis* subsp. *insidiosus* (**i**). AsR416 (I) and AsR36 (II) are tested proteins, As118 (III) and *B. subtilis* WB800 (IV) are controls. (**j**) Antibacterial effects of proteins screened by the *E. coli* expression system. AsRE67 (V) and AsRE39 (VI) proteins induced by IPTG, and AsRE67 (VII) without IPTG induction. (**k**) Antifungal effects of proteins screened by *B. subtilis* expression system. The *P. capsici* was inoculated in the center. AsR416 (I) and AsR36 (II) are tested proteins, and As118 (III) and *B. subtilis* WB800 (IV) are controls. Only the AsR416 protein had a significant effect against *P. capsici*. Experiments were repeated three times under the same conditions, and similar results were observed.

After the identification of clones producing intracellular toxicity against host cells (expressed candidate proteins played antimicrobial roles from inside of the cells), their extracellular antimicrobial activities were examined. In the case of *B. subtilis*, after the extraction of proteins from 48 selected clones, 25 proteins showed significant antimicrobial activities against Gram-positive and Gram-negative bacteria compared to controls, while others showed no effect (Fig. 2a–i, Table 1). Among the selected bacteria, *Clavibater michiganensis* subsp. michiganense, *C. fangii*, *C. michiganensis* subsp. insidiosus and *Rastonia solanacearum* were the plant pathogens, while *B. cereus* and *B. anthracis* were the human pathogenic bacteria. One clone showing no autolysis, named *As*118 (100 bp), was selected as control bacteria. Sequence analysis on NCBI indicated that 11 sequences showed no homology with known proteins and were considered to be novel (Table S1). In the case of *E. coli* clones, only one protein from one clone showed antibacterial activity against *E. coli* DE3, while the remaining 69 proteins did not show any antimicrobial effect (Fig. 2j). Antimicrobial activity against fungal pathogens was also evaluated, and *AsR*416 from the *B. subtilis* screening system showed maximum inhibition against *Phytophthora capsici* compared to *AsR*117 and *AsR*498, while other proteins did not show any significant inhibition (Fig. 2k). *AsR*416, *AsR*117 and *AsR*379 showed strong antimicrobial activities, but *AsR*416 was considered the best antimicrobial candidate gene, as it has shown strong antibacterial and antifungal activities (Table 1).

**Table 1:** Antimicrobial spectrum of *A. sativum* antimicrobial genes.

	Test genes	*WB800*	*AsR379*	*AsR117*	*AsR412*	*AsR416*	*AsR453*	*AsR36*	*AsR174*	*AsR864*	*AsR498*	*AsR845*	*AsR853*
Fungus	*Fusarium* spp	—	—	—	—	—	—	—	—	—	—	—	—
	*Botrytis cinerea*	—	—	—	—	—	—	—	—	—	—	—	—
	*P. capsici*	0.0 ± 0.00c	—	0.8 ± 0.06b	—	—	1.3 ± 0.25a	—	—	—	—	1.0 ± 0.10b	—
Gram-negative bacteria	*X. campestris* pv*. oryzicola*	—	—	—	—	—	—	—	—	—	—	—	—
	*A. tumefaciens*	—	—	—	—	—	—	—	—	—	—	—	—
	*E. coli* DE3	—	—	—	—	—	—	—	—	—	—	—	—
	*R. solanacearum*	0.9 ± 0.15f	2.1 ± 0.32a	1.6 ± 0.10b	1.7 ± 0.06b	2.1 ± 0.21a	1.3 ± 0.06cde	1.5 ± 0.06bc	1.2 ± 0.06ef	1.1 ± 0.10ef	1.9 ± 0.15a	1.4 ± 0.06bad	1.3 ± 0.15cde
Gram-positive bacteria	*C. fangii*	0.5 ± 0.15f	1.9 ± 0.15abc	1.6 ± 0.23bcd	1.6 ± 0.21bcd	2.1 ± 0.17a	1.6 ± 0.06bcd	1.2 ± 0.10e	1.2 ± 0.15e	1.2 ± 0.15e	2.0 ± 0.21ab	1.4 ± 0.28de	1.7 ± 0.21bcd
	*C. michiganensis* subsp.	0.0 ± 0.00e	1.7 ± 0.15a	1.6 ± 0.12ab	1.5 ± 0.10bc	1.8 ± 0.00a	1.5 ± 0.10bc	1.2 ± 0.06d	1.2 ± 0.15d	1.2 ± 0.10d	1.7 ± 0.15a	1.3 ± 0.21cd	1.6 ± 0.06ab
	*C. michiganensis* subsp.	0.0 ± 0.00e	1.9 ± 0.12a	1.4 ± 0.06bc	1.4 ± 0.12bc	1.9 ± 0.15a	1.2 ± 0.20cd	1.1 ± 0.20cd	1.0 ± 0.00d	1.4 ± 0.21bc	1.6 ± 0.40b	1.2 ± 0.06cd	1.4 ± 0.15bc
	*B. anthraci*	0.0 ± 0.00f	1.6 ± 0.10abc	1.6 ± 0.23abc	1.4 ± 0.15cde	1.8 ± 0.12a	1.3 ± 0.15de	1.4 ± 0.10cde	1.2 ± 0.10e	1.3 ± 0.06de	1.7 ± 0.15a	1.7 ± 0.15a	1.5 ± 0.06bcd
	*B. subtilis* 330-2	0.8 ± 0.15f	2.4 ± 0.21a	1.7 ± 0.06bc	1.4 ± 0.06de	2.3 ± 0.10a	1.5 ± 0.36cde	1.2 ± 0.06e	1.3 ± 0.00e	1.5 ± 0.12cde	2.0 ± 0.06b	1.7 ± 0.23cd	1.5 ± 0.06cde
	*B. cereus*	0.8 ± 0.23f	2.0 ± 0.35ab	1.7 ± 0.44abc	1.6 ± 0.26bc	2.2 ± 0.42a	1.4 ± 0.36bc	1.4 ± 0.20bc	1.3 ± 0.15cd	1.4 ± 0.12bc	1.7 ± 0.12abc	1.4 ± 0.26bc	1.2 ± 0.10cd
	*B. subtilis* 168	0.0 ± 0.00h	2.2 ± 0.06a	1.8 ± 0.10c	1.4 ± 0.15de	2.2 ± 0.17a	1.1 ± 0.10g	1.6 ± 0.10d	1.2 ± 0.12gf	1.3 ± 0.15ef	2.0 ± 0.10ab	1.9 ± 0.17bc	1.6 ± 0.15d
	*B. subtilis* WB800	0.0 ± 0.00c	1.3 ± 0.15a	0.6 ± 0.55b	0.9 ± 0.15ab	1.1 ± 0.15ab	0.9 ± 0.12ab	0.0 ± 0.00c	0.0 ± 0.00c	0.6 ± 0.55b	0.7 ± 0.64b	0.0 ± 0.00c	1.1 ± 0.15ab

— No inhibition; data presented here show average inhibition diameter (cm) and standard deviation. The results are the mean values from three independent experiments. Significance analysis was performed by SASS software, P ≤ 0.05.

Haemolytic activity test was performed to analyze the toxicity potential of 3 different proteins against mammalian cells. Haemolytic levels of 0 (negative control) and 100% (positive control) were determined in phosphate buffered saline (PBS) and 0.1% Triton X-100, respectively. The results revealed that a maximum concentration of 1000 µg/ml of these proteins showed nonsignificant haemolytic activity against sheep red blood cells, while lower concentrations did not show any haemolytic activity (Table S2). These results indicate that these proteins might have potential to be used in human medicines.

From these results, it can be concluded that the *B. subtilis* screening system is more efficient than the *E. coli* system, as many stable antimicrobial proteins were found from the *B. subtilis* expression system.

### The *B. subtilis* expression system facilitated the screening of antimicrobial genes from *P. ternata*

To validate the feasibility and efficiency of the *B. subtilis* expression system for the screening of antimicrobial genes, a *P. ternata* cDNA library was also constructed. From 2000 cDNA clones, 42 clones showing autolysis were screened out, and 23 clones showed antimicrobial activities. After sequencing, from NCBI analysis, 8 different sequences were found that are novel genes (Table S3). In a *Caenorhabditis elegans* repellence assay, tested proteins were shown to exhibit repellence to nematodes compared to controls. Microscopic analysis demonstrated that *C. elegans* did not exhibit active crawling, which was a clear indication of the toxicity of tested proteins (Fig. S2).

The *P. ternata* genes that suppressed the growth of bacteria and nematodes are listed in Table 2, and *PtR*280 and *PtR*743 showed significant levels of antibacterial activity. These results suggested that the *B. subtilis* expression system is a good choice for the screening of antimicrobial genes.

**Table 2:** Antibacterial spectrum of *P. ternata* antimicrobial genes.

	Test genes	*WB800*	*PtR1259*	*PtR280*	*PtR325*	*PtR743*	*PtR594*	*PtR840*	*PtR857*	*PtR1776*
Nematodes	*C. elegans*	—	0.06 ± 0.13b	−0.55 ± 0.02d	−0.50 ± 0.03d	−0.59 ± 0.04d	−0.25 ± 0.11c	0.23 ± 0.08a	−0.36 ± 0.05c	−0.50 ± 0.02d
Gram-negative bacteria	*X. campestris* pv*. oryzicola*	—	—	1.8 ± 0.15	—	—	—	—	—	—
	*A. tumefaciens*	—	—	—	—	—	—	—	—	—
	*E. coli* DE3	—	—	1.5 ± 0.20	—	—	—	—	—	—
	*R. solanacearum*	0.9 ± 0.20f	2.1 ± 0.15bc	1.8 ± 0.10de	1.6 ± 0.15e	2.7 ± 0.20a	2.0 ± 0.06cd	2.9 ± 0.25a	2.8 ± 0.38a	2.4 ± 0.06b
Gram-positive bacteria	*C. michiganensis* subsp*. michiganense*	0.0 ± 0.00d	2.1 ± 0.15ab	1.6 ± 0.15c	1.9 ± 0.06b	2.2 ± 0.23a	1.5 ± 0.10c	2.2 ± 0.15a	1.9 ± 0.17b	2.1 ± 0.15ab
	*B. anthraci*	0.0 ± 0.00c	1.5 ± 0.23ab	1.8 ± 0.17a	1.5 ± 0.20ab	1.4 ± 0.06b	1.8 ± 0.21a	1.4 ± 0.15b	1.4 ± 0.15b	1.6 ± 0.15ab
	*B. subtilis* 330-2	0.8 ± 0.10e	2.0 ± 0.15b	2.0 ± 0.12b	1.7 ± 0.15c	3.2 ± 0.26a	1.8 ± 0.12bc	2.0±0.15b	1.6 ± 0.10c	1.1 ± 0.06d
	*B. cereus*	0.7 ± 0.12d	1.4 ± 0.20abc	1.6 ± 0.10a	1.3 ± 0.15bc	1.3 ± 0.20bc	1.6 ± 0.15a	1.3 ± 0.10bc	1.2±0.15c	1.4 ± 0.25abc
	*B. subtilis* 168	0.0 ± 0.00e	0.0 ± 0.00e	2.0 ± 0.15a	1.5 ± 0.10d	1.9 ± 0.17ab	1.5±0.15cd	1.7 ± 0.12bc	1.9 ± 0.15ab	1.8 ± 0.10ab
	*B. subtilis* WB800	0.0 ± 0.00c	0.0 ± 0.00c	0.8 ± 0.06b	1.2 ± 0.15a	1.3 ± 0.06a	1.2 ± 0.20a	1.1 ± 0.10a	0.0 ± 0.00c	1.3 ± 0.17a

— No inhibition; data presented here show the average inhibition diameter (cm) and standard deviation. The results are the mean values from three independent experiments. Significance analysis was performed by SASS software, P ≤ 0.05.

### Bioinformatics analysis of the isolated antimicrobial proteins

Understanding the bioinformatics aspects of proteins is of great importance to understand the relationship between structure and function, and it is also important for optimization and modification of the proteins. Translation analysis of *A. sativum* and *P. ternata* cDNA sequences revealed that these sequences consisted of 10 to 75 amino acids ranging from short peptides to complete proteins (Table 3). On the basis of the structure-function relationship of proteins, secondary structures of these proteins were predicted using bioinformatics tools and divided into four classes: (1) α-helices, (2) β-strands, (3) both α-helix and β-strand or (4) extended (non-αβ). According to our analysis, 4 out of the 19 antimicrobial proteins belonged to the first group, 7 belonged to the second group and all were amphipathic, while 8 belonged to the third group and most of these were hydrophobic amino acids. From the results (Table 3), we speculated that different structures might lead to different antibacterial activities. We found that several antimicrobial proteins containing both α-helices and β-strands have shown significant levels of antibacterial activities, including AsR416, AsR117 and AsR379 from *A. sativum*, while PtR280 and PtR743 from *P. ternata* and proteins containing α-helices usually showed more consistently antibacterial activities. Seven out of the 19 sequences had disulfide bonds. The PtR280 protein with good antimicrobial activities from the *P. ternata* cDNA library had 3 disulfide bonds.

**Table 3:** Bioinformatics prediction of antimicrobial proteins.

	Protein name	AA No.	S-S No.	Secondary Structure	pI	MV	GRAVY
*A. sativum* antimicrobial protein	AsR117	34	1	α-helix β-strand	6.90	3959.75	0.712
	AsR416	34	1	α-helix β-strand	8.06	3799.52	0.850
	AsR379	20	1	α-helix β-strand	6.73	2379.86	0.895
	AsR845	25	0	α-helix β-strand	5.99	3064.60	0.632
	AsR36	18	0	α-helix	8.66	2209.71	0.589
	AsR412	18	0	α-helix	9.31	2307.70	−0.356
	AsR853	13	0	α-helix	3.80	1538.74	0.415
	AsR864	16	0	β-strand	3.71	1887.13	0.212
	AsR453	18	1	β-strand	8.07	2048.41	0.261
	AsR174	13	0	β-strand	9.19	1722.04	−0.046
	AsR498	10	0	β-strand	4.00	1201.36	0.200
*P. ternate* antimicrobial proteins	PtR1259	29	1	α-helix β-strand	4.14	2954.38	0.941
	PtR280	75	3	α-helix β-strand	8.89	8318.82	0.385
	PtR325	18	0	α-helix β-strand	6.05	2227.61	−0.017
	PtR743	17	0	α-helix β-strand	3.57	1938.31	1.559
	PtR594	21	0	α-helix	5.98	2633.04	0.105
	PtR840	29	0	β-strand	4.21	3249.60	−0.003
	PtR857	71	1	β-strand	9.26	8507.90	0.300
	PtR1776	17	0	β-strand	4.35	1806.02	0.288

AA No.: number of amino acids, GRAVY: grand average of hydropathicity, pI: theoretical pI, MV: molecular weight, S-S No.: number of cysteine disulfides.

### Antimicrobial proteins were stable at high temperature ranges

**Figure 3:**
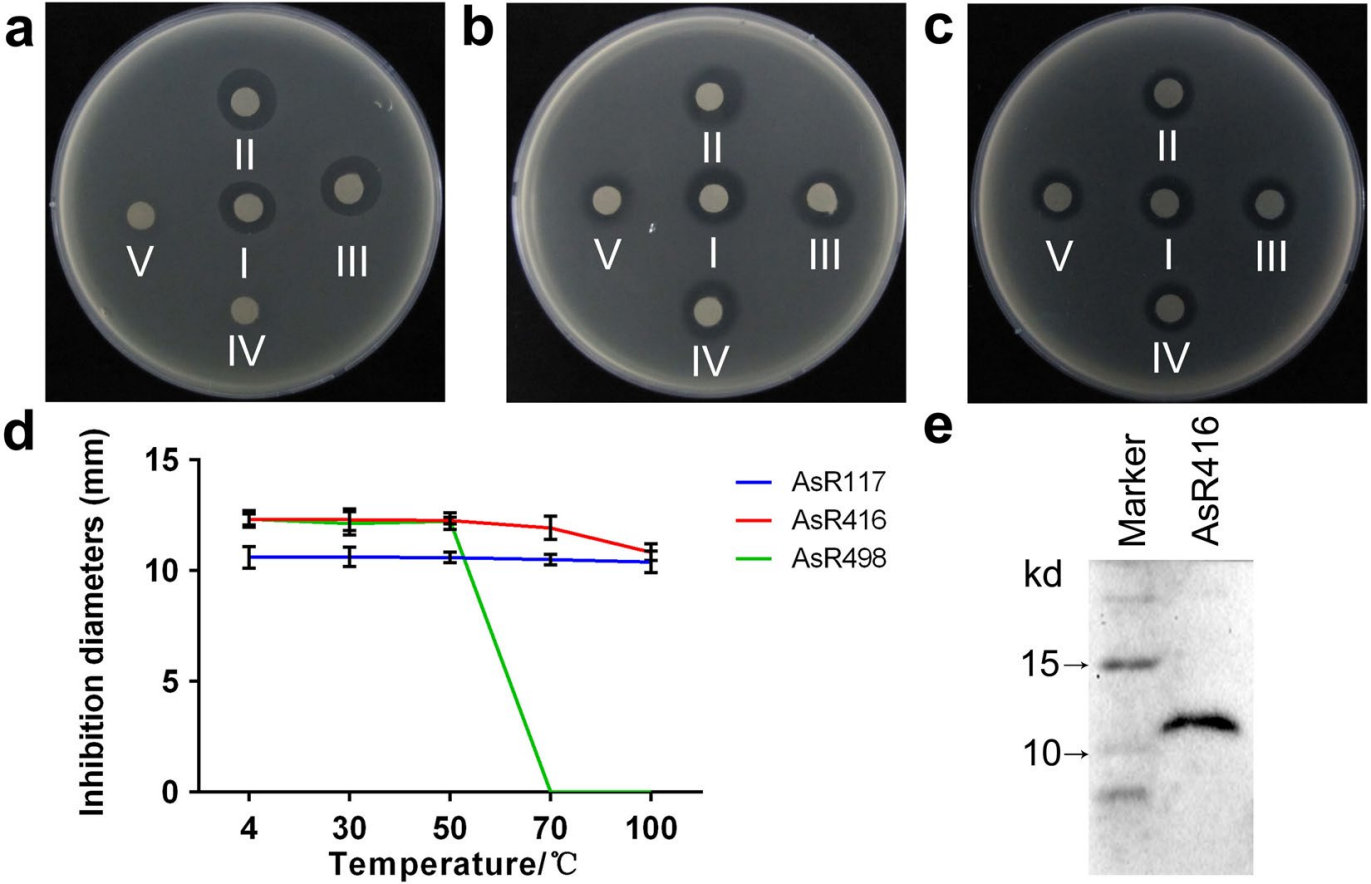
Protein thermal stability. AsR498 (**a**), AsR416 (**b**) and AsR117 (**c**) were confronted with *B. subtilis* 168 (background indicator bacteria) after heating at 4 °C (I), 30 °C (II), 50 °C (III), 70 °C (IV), and 100 °C (V) for 15 minutes. (**d**) Inhibition diameters of 3 proteins with temperature curves. Vertical bars shows SD. Data are the mean values from three individual experiments. (**e**) Western blot confirmed the expression of the purified *AsR*416 gene. The full-length blot was presented in Supplementary Fig. S3. Experiments were repeated three times under the same conditions, and similar results were observed.

Three different proteins, AsR117, AsR416 and AsR498, that had demonstrated significant antimicrobial activities were selected and heated at 4 °C, 30 °C, 50 °C, 70 °C and 100 °C for 15 minutes. AsR117 and AsR416 exhibited stable antibacterial activities at all high temperature ranges, unlike AsR498, which reduced its antibacterial activity after 50 °C (Fig. 3a–d). From these results, it was considered that some of these proteins share the thermal stable common characteristic with known antimicrobial peptides and might have potential to be used in clinical medicines or processed into antimicrobial agents after further required investigations.

### Effect of AsR416 protein on cell membrane integrity

**Figure 4:**
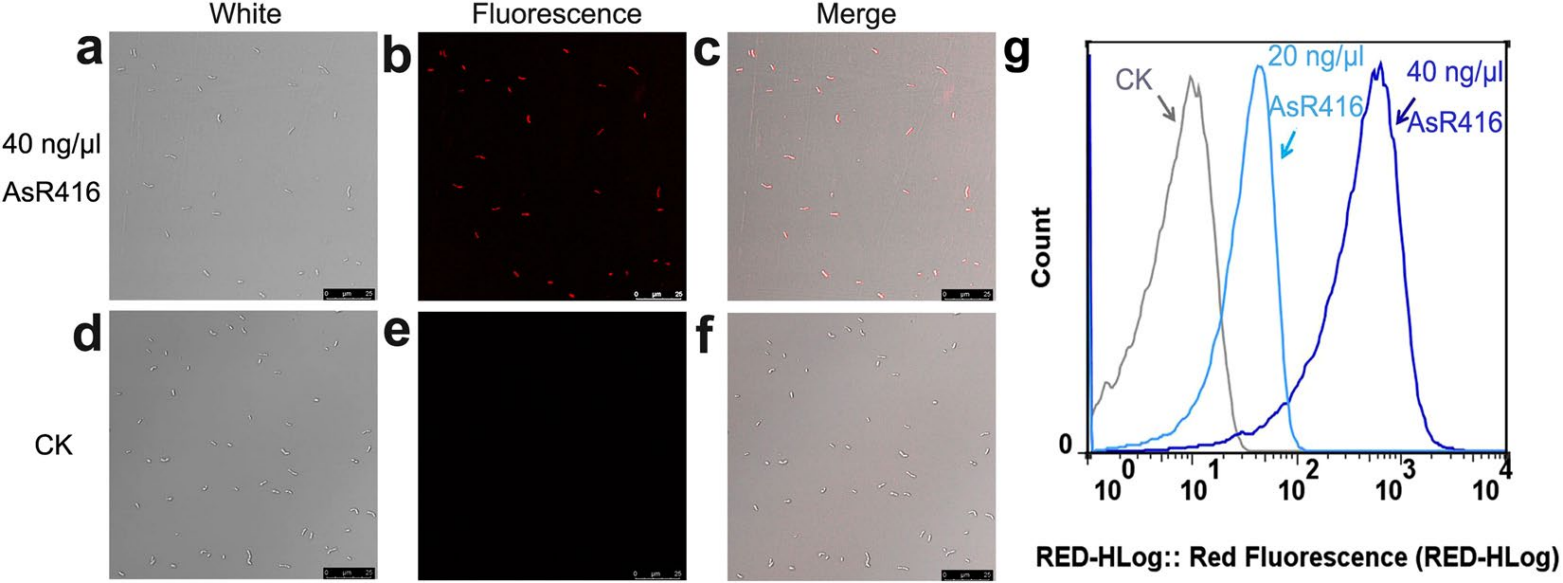
The PI uptake assay. (**a–f**) Confocal laser scanning microscopy analysis of PI staining. The *B. subtilis* 168 cells (1 × 10^8^ CFU/ml) incubated with AsR416 protein (**a–c**) and PBS buffer without test protein (**d–f**). The *B. subtilis* 168 cells under white light (**a,d**) and under fluorescence (**b,e**). (**c**) Combination of a and b. (**f**) Combination of d and e. (**g**) Flow cytometry analysis of PI staining. The *B. subtilis* 168 cells incubated with PBS buffer (control) and AsR416 and observed under FACSVerse machine. The x-axis shows the relative fluorescence intensity. Experiments were repeated three times under the same conditions, and similar results were observed.

The protein from the *AsR*416 gene was purified using His-tag, and expression was verified by western blot, which revealed that the mass of recombinant protein was approximately 12 kD (Figs 3e and S3). To further explore the effect of AsR416 on the integrity of the cell membrane, PI staining was performed. The PI is a DNA-intercalating fluorescent dye that can bind to DNA through the compromised cell membrane. The results of confocal laser scanning microscopy showed that almost all bacteria cells treated with AsR416 were stained with PI and exhibited red fluorescence (Fig. 4a–c). Striking contrast was observed without red fluorescence in the control in which PI was incubated with bacteria without test protein expression (Fig. 4d–f). Flow cytometry results have shown that after treatment with PBS buffer (control), 20 ng/μl AsR416 and 40 ng/μl AsR416, 0.01%, 61.03% and 93.97%, respectively, of *B. subtilis* 168 cells emitted fluorescence (Fig. 4g). These findings revealed that the membrane integrity of the bacteria cells was destroyed by AsR416 protein and the percentage of cell membrane damage increases with increasing protein concentration.

### Purified proteins inhibited the infection of *P. capsici*

**Figure 5:**
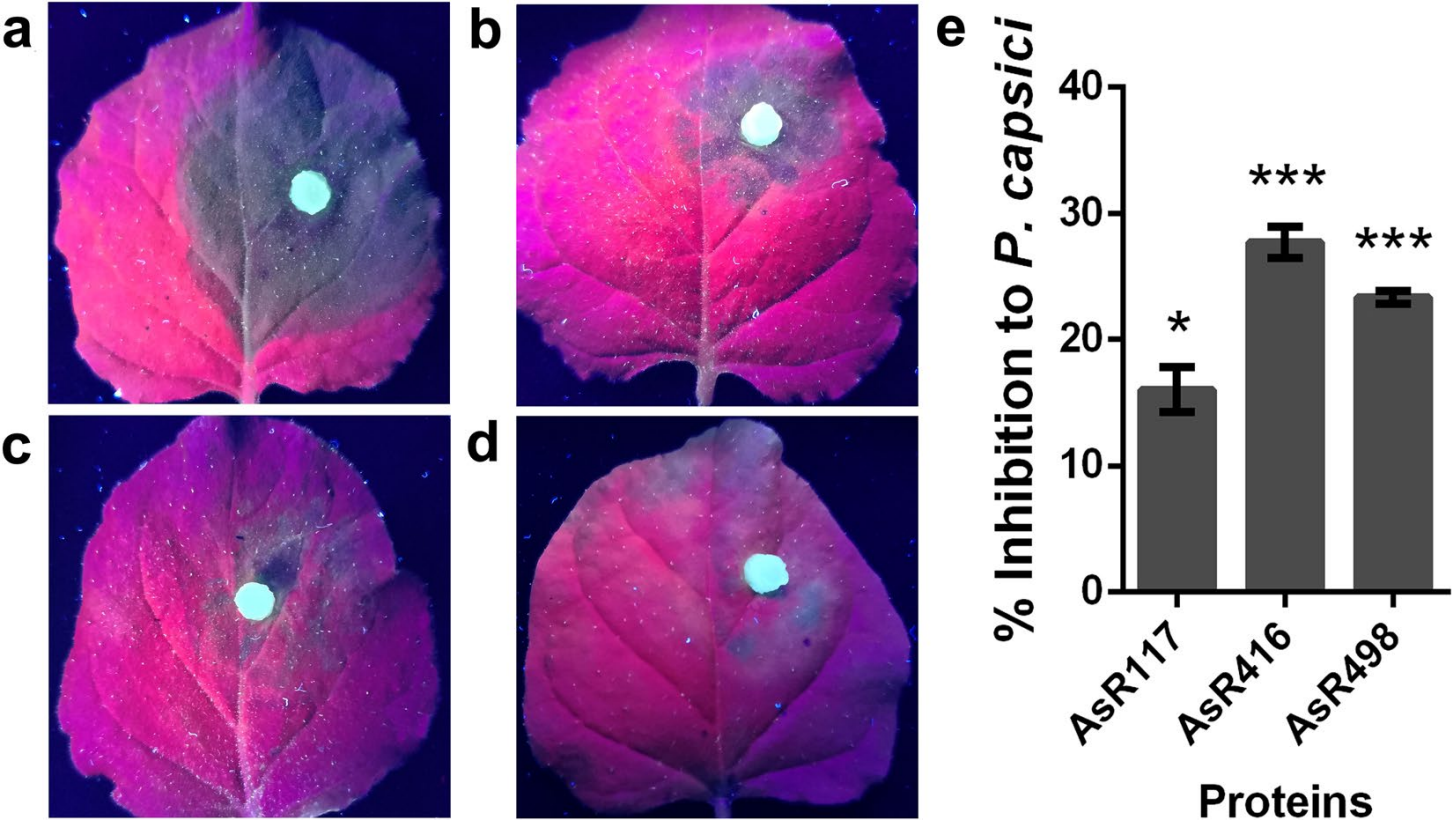
Percent inhibition of different proteins against *P. capsici*. Leaves after treatment with purified proteins of AsR498 (**b**), AsR416 (**c**) and AsR117 (**d**) at a concentration of 30 ng/μl. A disc of *P. capsici* was placed on tobacco leaves at 25 °C in darkness and humidity; lesion diameters were measured after 48 h. As118 (**a**) was used as control. Pictures were taken under UV light. (**e**) Percent inhibition against *P. capsici*. Vertical bars show SD. Data are the mean values from three individual experiments. Significance analysis was performed using the t-test. *p < 0.05 and **p < 0.01.

A layer of the purified peptides AsR117, AsR416, AsR498 and As118 was spread over detached tobacco leaves and then inoculated with *P. capsici*. All leaves treated with peptides significantly inhibited the *P. capsici* infection compared to control (Fig. 5a–d). The percent inhibition shown by AsR416 was the highest, while the minimum percent inhibition was shown by AsR117 (Fig. 5e). This provides a good theoretical basis for the future practical application of antimicrobial peptides.

### Secreted antimicrobial peptides/proteins inhibited the growth of pathogenic bacteria in soil

The *B. subtilis* strains harboring *AsR*117, *AsR*498 and *AsR*416 genes were selected to analyze their efficacy against the pathogenic bacteria *C. michiganensis* subsp*. insidiosus* and *R. solanacearum*. For this purpose, *As*118 (control), *AsR*117, *AsR*498 and *AsR*416 strains were cultured for two days and applied in sterilized soil. Two days later, test pathogenic bacteria were added to the soil. Soil samples were collected at 0-, 2- and 4-day intervals. From the results, it was observed that at the 4^th^ day of inoculation, amounts of *C. michiganensis* subsp*. insidiosus* and *R. solanacearum* were significantly reduced compared to control (Fig. S4). From these results, we concluded that our newly identified antimicrobial genes with their encoded proteins inhibited pathogenic bacteria in soil conditions.

## Discussion

In view of the bottleneck problem of finding new classes of antimicrobial genes, we constructed *A. sativum* cDNA libraries by using two different expression systems, *B. subtilis* and *E. coli*. By comparing their screening efficiency and antimicrobial activities, we concluded that the *B. subtilis* expression system is more suitable for screening antimicrobial peptides/proteins. The 19 novel genes identified by the *B. subtilis* expression system had broad antimicrobial activities and thermal stable characteristics and showed no haemolytic activities, supporting their potential use in clinical medicines or the development of antimicrobial biocontrol agents. This research has opened an avenue for researchers to approach a new comprehensive strategy for the identification of antimicrobial genes, which can play a role in the betterment of mankind by treating different diseases in a cost-effective way.

The two basic differences between *B. subtilis* and *E. coli* expression systems define their antimicrobial gene screening efficiency. The first difference is the host cell; the screening indicator of both systems is protein toxicity or antimicrobial roles against host cells. The *B. subtilis* has a natural ability to secrete proteins into their environment, often at high concentrations^33^, so its secretion characteristic reduces the toxicity of host cells. On the other hand, the *E. coli* system secretes proteins into their periplasmic space, resulting in toxic protein accumulation in the host cells, which leads to the bursting and complete death of cells, so it is difficult to obtain a high abundance of target proteins. Another difference is the expression vector, as the *B. subtilis* system uses the constitutive expression vector pBE-S, while the *E. coli* system uses the inducible expression vector pET-22b. Our results showed that the *B. subtilis* system is more efficient because it had lower initial screening numbers but successfully screened more antimicrobial genes. The *E. coli* expression system needs to be induced by IPTG, so there is a magnitude gap between constitutive expression system and inducible expression system that cannot be replaced. Although single *E. coli* colonies stained by trypan blue were considered to have antibacterial ability, it was reported in previous studies that in addition to dead or damaged cells, normal cells can also be stained due to increased cell membrane permeability caused by trypan blue staining^14^.

Furthermore, the results in Tables 1 and 2 show that our antibacterial proteins have stronger activities against Gram-positive bacteria. The *B. subtilis* is a Gram-positive bacterium, while *E. coli* is a Gram-negative bacterium. Therefore, *B. subtilis* is more efficient for the screening of antimicrobial genes against Gram-positive bacteria, whereas *E. coli* is more likely to screen antimicrobial genes that are effective against Gram-negative bacteria. Currently, there are 426 peptides reported against Gram-positive bacteria but only 202 peptides against Gram-negative bacteria reported in the antimicrobial peptide database^34^. Hundreds of Gram-positive pathogens not only infect plants but also endanger the health of humans and animals; for example, *B. anthracis* can cause human anthrax^35^, *B. cereus* is reported to cause food poisoning, eye infection, fulminant sepsis and devastating central nervous system infections^36^, and *C. michiganensis* can infect *Solanum lycopersicum*, *S. tuberosum* and *Medicago sativa*^37^. Additionally, our peptides/proteins have resistance to fungus, can repel nematodes and show no haemolytic activities. Previous researchers have found peptides/proteins with wound healing, anti-HIV, anticancer and antilung disease activity^38–41^, which illustrates the importance of antimicrobial peptides/proteins in human health. Our system provides an avenue to the study diversity and similarity of antimicrobial peptides/proteins and introduce a strategy for the screening of antimicrobial peptides/proteins, not only specific to the elimination of bacteria that have become resistant against existing antibiotics but also against plant and human pathogenic microorganisms.

Moreover, *B. subtilis* has another advantage over *E. coli* in that members of *Bacillus* spp can be used as biological control agents^42^. From the results (Fig. S4), it has been observed that our functional genes with encoded proteins can be used directly as biocontrol agents in soil to inhibit the growth of pathogenic bacteria. A number of experiments have been conducted in which peptides were applied to plants to increase plant disease resistance^8^. A previous study reported that a plant immunity-inducing protein PeaT1 recombinant plasmid transformed into *B. subtilis* can increase the production and drought tolerance potential of wheat crops^43^. It is also reported that *B. subtilis* can induce systemic resistance to promote the health and growth of plants^44,45^. Interestingly, genes screened in our case were already expressed in the *B. subtilis* expression system, which excludes the need for cloning these genes into another expression vector and enhances the value of these genes as biocontrol agents. Although the colony autolysis phenomenon was observed on solid culture medium plates, colonies could grow in liquid culture medium. It is an important characteristic of these antimicrobial genes to be considered as biocontrol agents.

According to bioinformatics prediction, most of our antimicrobial proteins have a significant proportion of hydrophobic residues with cationic charges, which facilitate interaction with the fatty acyl chains and allow them to promote selectivity for negatively charged microbial cytoplasmic membranes over zwitterionic MAM Malian membranes^46^. Our peptides/proteins are mostly composed of α-helices, which is consistent with the fact that most AMPs are composed of α-helical structures^47,48^. Previous studies have reported that different secondary structures lead to different antibacterial activities^49^. Combining the results of Tables 1, 2 and 3, we assumed that the antimicrobial peptides that contain both α-helices and β-strands may exhibit better antibacterial activities. The molecular weight of AsR416 protein was approximately 12 kD after western blot (Fig. 3e), while The sequence translation revealed that its estimated size should be approximately 3.8 kD (Table 3), which is 8.2 kD less than the molecular weight of AsR416 observed after western bot, so it is assumed that this extra 8.2 kD molecular weight is the result of additional sequences from the vector and His-tag used to analyze the protein expression. Many researchers have reported that the molecular weight of a His-tag fusion protein is larger than the expected mass after SDS-PAGE^50–52^.

Antibiotics usually have specific sites of action, e.g., tetracycline inhibits ribosomal 30 S subunit^53^, 4-quinolones antibiotics inhibit DNA gyrase^54^, and some antibiotics act on protein synthesis or folding, or cell wall synthesis. In contrast to most antibiotics, the majority of antimicrobial proteins act on the plasma membrane of bacteria or other generalized targets^6^. This specific mechanism of antimicrobial proteins makes it almost impossible for microbes to develop resistance through gene mutation^55^. Flow cytometry and confocal laser scanning microscopy assay demonstrated that our proteins disrupt the cell membranes of bacteria (Fig. 4). Here, our proteins can induce PI, a membrane-impermeable fluorescent dye that can bind to genomic DNA, to enter the cell, which is possible evidence for our protein to target the cell membrane. Of cause, further research is necessary to provide direct evidence at the molecular level.

In conclusion, we designed a novel, sensitive and high-throughput strategy to isolate antimicrobial genes. In brief, this method transforms a cDNA library into the *B. subtilis* expression system and allows thousands of expressed proteins to work from inside of the *B. subtilis* cells (very sensitive and high throughput). We used the *B. subtilis* cell as a target indicator and screened antibacterial proteins. Our findings not only have provided 19 novel broad-spectrum antimicrobial genes but also have indicated that plant and human pathogenic bacteria, fungi and even cancer cells might be taken as the indicator target cells for screening specific antimicrobial genes against plant and human pathogenic bacteria, fungi and cancer cells.

## Material and Methods

### Maintenance of plant and pathogen cultures

*Pinellia ternata* and *A. sativum* were grown in a controlled growth chamber at 26 °C/20 °C and 14 h/10 h of light/dark conditions. *Nicotiana benthamiana* seeds were germinated on nutrient soil and transplanted individually in pots under controlled growth conditions of 24–28 °C and 16 h/8 h light and dark intervals. *Rhizoctonia solani* was grown on PDA (Potato dextrose agar) media at 28 °C. *Botrytis cinerea* and *Fusarium* spp were cultured on PDA plates at 25 °C. *Phytophthora capsici* (Li263) was maintained on V8 (100 ml V8 juice, 1 g CaCO_3_ per liter distilled water) media at 25 °C under dark conditions. *Erwinia carotovora* subsp. *carotovora* was maintained on LB (10 g tryptone, 5 g yeast extract, and 10 g NaCl per liter distilled water) at 28 °C. The *B. subtilis* WB800*, B. subtilis* 168*, B. cereus, B. subtilis* 330-2*, B. anthraci, C. michiganensis* subsp. *michiganense*, *R. solanacearum, E. coli* DE3, and *X. campestris* pv. oryzicola were maintained on LB media at 37 °C. The *A. tumefaciens* was maintained on LB media at 28 °C. *Clavibater fangii* and *C. michiganensis* subsp*. insidiosus* were grown on MS45 media (protein 10 g, yeast extract 5 g, malt extract 5 g, casein amino acid 5 g, beef extract 2 g, glycerin 2 g, Tween 80 50 mg, magnesium sulfate heptahydrate 1 g, and agar 15 g, constant volume to 1 l, pH 7.2) at 30 °C and 28 °C, respectively. *Caenorhabditis elegans* wild-type strain N2 was maintained on NGM (nematode growth media: protein 2.5 g, sodium chloride 3 g, and agar powder 15 g, constant volume to 1 L, 200 µl of magnesium sulfate, 200 µl of calcium chloride, 200 µl of cholesterol, and 5 ml of potassium phosphate were added before making a solid plate) at 25 °C.

### Construction of cDNA libraries

Construction of cDNA libraries followed the general scheme: extraction of RNA, purification of mRNA, cDNA synthesis, ligation with vector and transformation. Detailed construction protocols can be found in Supplementary Methods. Primers for construction of two cDNA libraries as well as pictures of RNA, mRNA and cDNA can be found in Supplementary Tables S4 and S5 and Supplementary Fig. S5.

### cDNA library quality assessment

Primary library titers and recombination rates were calculated as described^32^. For insert size detection, 100 single colonies were randomly picked from the library, and colony PCR was performed under following conditions: 95 °C 5 minutes; 95 °C for 30 s, 55 °C for 30 s, 72 °C for 50 s (30 cycles) and 72 °C for 8 minutes. Quality was assessed by gel electrophoresis and sequence analysis.

### Screening of cDNA libraries

For the *B. subtilis* secretory protein expression system, single clones on the plates were picked with a toothpick and cultured at 37 °C and 180 rpm for 5–8 h in LB medium containing kanamycin. From each tube, 2 μl bacteria was taken and dropped onto the plate with 1 cm lattice. Bacterial growth was observed after every 12 h until 60 h, and the numbers of lysed bacteria were recorded. Plasmids were extracted and transformed into *B. subtilis* WB800 to verify the stability of autolysis.

For *E. coli* expression systems, screening was performed using a vital dye-staining method with slight changes^56^. Detailed screening protocols can be found in Supplementary Methods.

### Protein extraction of candidate genes

A candidate protein from the *B. subtilis* secretory protein expression system was extracted by the ammonium sulfate precipitation method^57^. Candidate proteins from the *E. coli* system were extracted by the freezing and thawing method using liquid nitrogen. Detailed extraction protocols for both kinds of proteins can be found in Supplementary Methods.

### Evaluation of proteins against pathogenic microbes

For the antibacterial bioassays, a filter paper pairing method was used, and proteins were dropped on plates containing indicator bacteria. For the heated-protein antibacterial bioassay, the basic steps were similar. Detailed protocols can be found in Supplementary Methods.

The antifungal assay was performed according to the protocol described by the method of Xiao *et al*.^58^. Detached tobacco leaves were smeared with a layer of 30 ng/μl protein, and the hyphae block was placed on it after 5–8 h and protected from light in an incubator at 25 °C and 90% humidity. After 48 h of incubation, inhibition zones were measured. Percent inhibition was calculated using the following formula: 100 × [(average colony diameter of control - average colony diameter of treatment)/average colony diameter of control]. The nematode repellence assay was assessed as described^59^. On the NGM plate, nematodes were placed in the middle of the test protein and the control protein, and nematode distribution was observed continuously over 6–12 h.

### Haemolysis assays

After the sheep red blood cells were incubated with the protein, percent haemolysis was calculated using the formula: percent haemolysis = [(Abs540 nm in the peptide solution − Abs540 nm in PBS)/(Abs540 nm in 0.1% Triton X-100 − Abs540 nm in PBS)] × 100. Detailed steps were as described in^60^ and in Supplementary Methods.

### Scanning electron microscopy

Bacterial cell surface morphology was observed through a scanning electron microscope (SEM) as described^61^. Specific steps for bacterial culture, sample fixation, and dehydration are described in Supplementary Methods.

### Sequence prediction

Protein sequences were translated with the EMBOSS Programs (https://www.ebi.ac.uk/Tools/emboss/). The secondary structure of the antimicrobial protein was predicted by the PSIPRED team’s website (http://bioinf.cs.ucl.ac.uk/psipred/). DISULFIND was used to predict whether the protein contained disulfide bonds (http://disulfind.dsi.unifi.it/)^62^. Online ExPASy tools were used to predict the pI, molecular mass and grand average of hydropathicity of the predicted proteins (http://expasy.org/tools).

### Protein purification and western blot

Recombinant proteins AsR416, AsR117 and AsR498 were isolated according to the protocol using the Ni-NTA His Bind Resin kit. The 40 µl of purified AsR416 was boiled in water for 10 minutes with 10 µl of 6x protein loading buffer. Then, 15 µl of mixed samples were separated on a polyacrylamide gel using the Trisine-SDS-PAGE kit. The western blot procedure was performed as described^51^. Western blot was performed as described in Supplementary Methods.

### Confocal laser scanning fluorescence microscopy and flow cytometry analysis

For confocal microscopy, bacteria were prepared as described by Xie *et al*.^63^. Cells in the mid-log phase (1 × 10^8^ CFU/ml) were collected by centrifugation, washed three times with PBS, and then treated with PBS, 20 ng/μl or 40 ng/μl AsR416 protein at 37 °C for 1.5 h. After treatment, cells were washed twice, and the PI concentration was fixed at 10 μg/ml for 30 minutes in dark at 4 °C and immobilized on a glass slide. Confocal microscopy images were taken using a Leica microsystems CMS GmbH TCS SP8 (Leica, Germany). For flow cytometry, bacteria were prepared using the above methods. Fluorescence was measured by a FACSVerse machine (BD, USA) according to method described by Lee *et al*.^64^, and the data were analyzed by Flowjo.7.6.1.Min software (BD, USA).

### Detection of pathogenic bacterial density in soil

To detect the density of *C. michiganensis* subsp*. insidiosus* and *R. solanacearum* in the soil against the application of the antibacterial genes *As*118 (control), *AsR*416, *AsR*498 and *AsR*117, sterilized equal-sized pots were filled with an equal amount of sterilized soil, and antibacterial recombinant strains were applied. After two days of incubation, calculated concentrations of preactivated pathogenic bacteria were inoculated in the soil. At 0, 2 and 4 days, 10 g of soil samples were collected, dissolved in 100 ml of sterile water, and shaken at 150 rpm for 30 minutes. Supernatant was collected and serially diluted to spread over NA plates. The number of colonies on the plates was counted after 12 h, and the density of pathogenic bacteria in soil was calculated as CFU.

## Electronic supplementary material

Supporting Information
