# Supplementary material for: Antimicrobial genes from Allium sativum and Pinellia ternata revealed by a Bacillus subtilis expression system
Source: Sci Rep. 2018 Sep 28;8:14514. doi: 10.1038/s41598-018-32852-x (PMC6162269; doi:10.1038/s41598-018-32852-x)
Supplement: Supplementary file 1 — Supporting Information [file 41598_2018_32852_MOESM1_ESM.pdf]

**Supporting Information**

# Antimicrobial genes from *Allium sativum* and *Pinellia ternata* revealed by a *Bacillus subtilis* expression system

Xi Kong, Mei Yang, Hafiz Muhammad Khalid Abbas, Jia Wu, Mengge Li, Wubei Dong*

Department of Plant Pathology, College of Plant Science and Technology and the Key Lab of Crop Disease Monitoring & Safety Control in Hubei Province, Huazhong Agricultural University, Wuhan, Hubei Province, 430070, China.

*Corresponding author

E-mail: dwb@mail.hzau.edu.cn

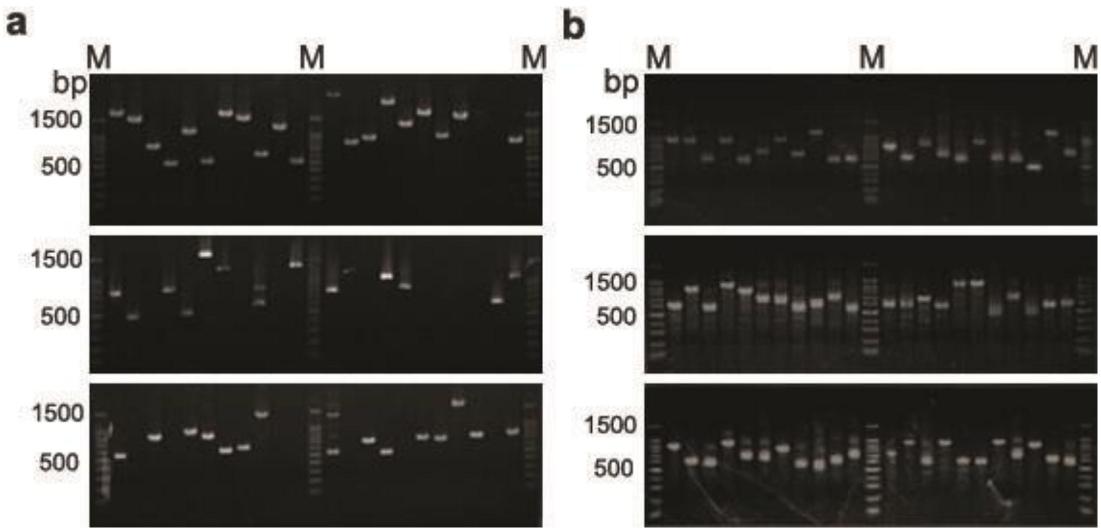


**Figure S1.** ***Allium sativum*** **cDNA library insert sizes.** (**a**) The cDNA library of *E. coli* expression system was inserted between 500 – 2000 bp. (**b**) The cDNA library of *Bacillus subtilis* expression system was inserted between 600 – 1500 bp.

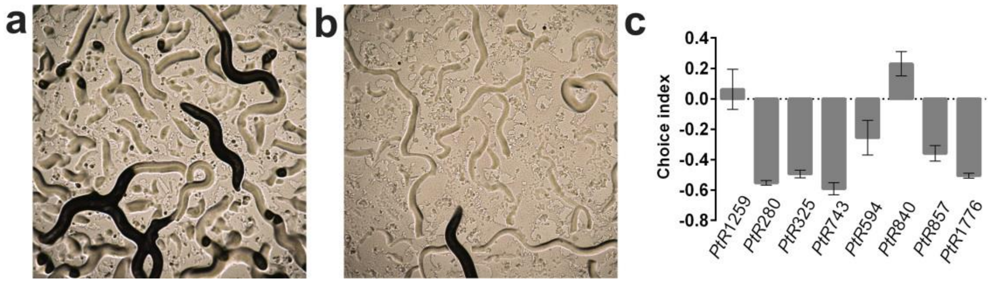


**Figure S2. Nematicidal activities of test proteins.** (**a-b**) Nematode repellence assay. The nematodes of the fourth stage larvae (L4) were placed on the NGM medium (nematode growth medium) between the test protein and the control protein (the distance was very important and must be equally spaced), and the condition of the nematode was continuously observed under microscope after 6 - 12 h. (**a**) More nematode individuals were present far away from the antimicrobial protein, (**b**) less number of nematodes within the range of the resistant protein. (**c**) CI (choice index) = (number of nematodes in lawn A) - number of nematodes in lawn B) / total number of nematodes. CI value < 0, nematode preferred strain B; CI value > 0, nematodes preferred strain A; CI value = 0, no preference for nematodes. Strain B stands for 11 different antimicrobial strains, including *PtR*1259, *PtR*280, *PtR*325, *PtR*743, *PtR*594, *PtR*840, *PtR*857 and *PtR*1776, and strain A is the control. Experiments were repeated three times under the same conditions and similar results were observed. Vertical bars showed SD.

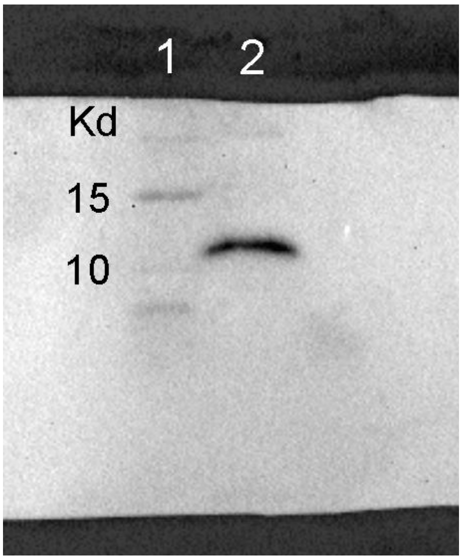


**Figure S3. Original full-length western blot of the AsR416 protein.** The color pre-staining ultra-low molecular weight (1.7 - 40 Kd) maker showed in lane 1 and the AsR416 protein in lane 2.

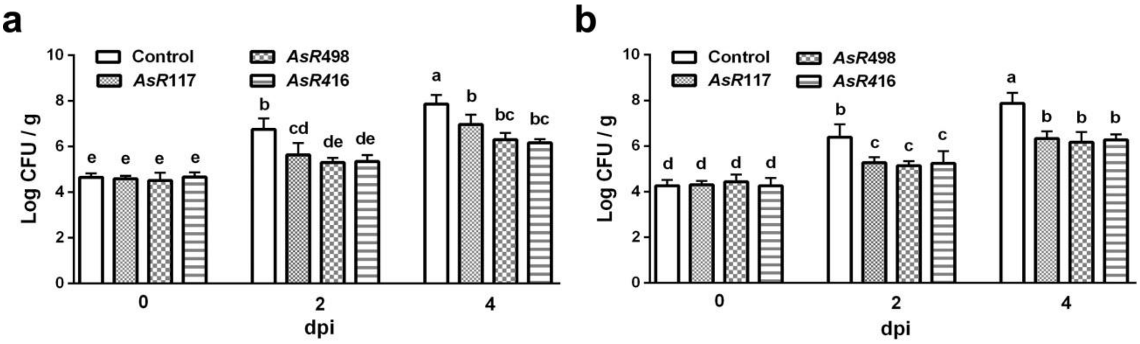


**Figure S4. Inhibition of pathogenic bacteria by *B. subtilis* strains harboring antimicrobial genes under soil conditions.** Weighed amount of *B. subtilis* treated soil was shaken in sterile water at 37 ºC. Supernatant was collected and gradient dilutions of the supernatant were spread over NA plates. Number of colonies were counted on each plate to calculate the colony-forming units (CFU). (**a**) *As*118 (control), *AsR*117, *AsR*498 and *AsR*416 against *Rastonia solanacearum*. (**b**) *As*118 (control), *AsR*117, *AsR*498 and *AsR*416 against *Clavibater michiganensis* subsp*. insidiosus*. Vertical bars are showed SD. Results are the mean values from three individual experiments. Significance analysis was performed by SASS software, P≤0.05.

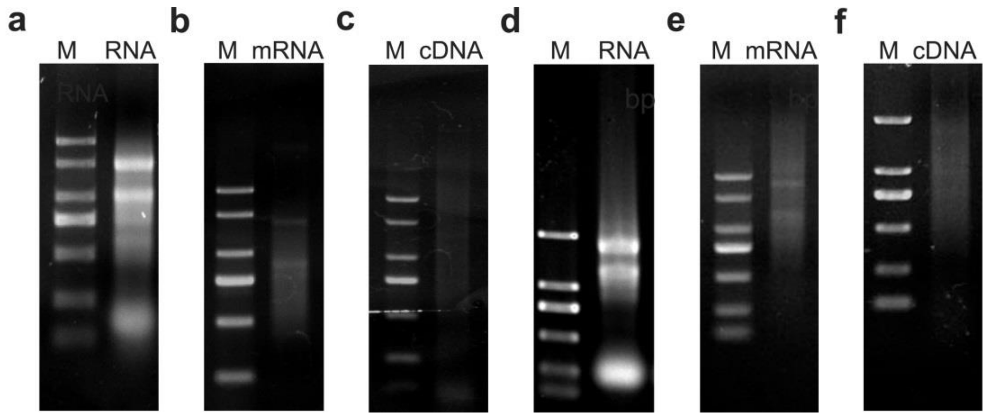


**Figure S5. Quality of RNA and cDNA in cDNA library construction.** The quality of total RNA (**a**), mRNA (**b**), and cDNA (**c**) of *A. sativum* cDNA library. The quality of total RNA (d), mRNA (e), and cDNA (f) of *P. ternata* cDNA library. Marker of (**a-c**) and (**e**) is the DL2000 bp marker. Marker of (**d**) and (**f**) is the 100 bp marker.

**Table S1.** ***Allium sativum*** **antibacterial protein amino acid sequences**

| Protein name | Amino acid sequence | Amino acid number |
|---|---|---|
| AsR117 | YMYRVCVSLFNGMDHIAVMYVYVCLHKGMDSSLL | 34 |
| AsR416 | YVLLLLIACFGMNCLLWNRQRVSDIGGWGSVTSL | 34 |
| AsR379 | KYSNVLELCHVISWVIIYCP | 20 |
| AsR845 | FTFCFLSFLCDGEFHLCYKMYHIAQ | 25 |
| AsR36 | FLLFISCCFSKKLEKWCN | 18 |
| AsR412 | YNNIELCLRNNFIKRHFL | 18 |
| AsR853 | SNCWNNDIFSLLI | 13 |
| AsR864 | YLEFLDLDCTKILDDA | 16 |
| AsR453 | IVIVCSESEGINIRCRRT | 18 |
| AsR174 | NYIFPRIRTCYYL | 13 |
| AsR498 | NNELYMFVAT | 10 |

**Table S2. Haemolytic activity of extracted proteins against sheep red blood cells**

| Treatments | Percentage haemolysis at different concentrations （μg/ml） | | | |
|---|---|---|---|---|
| | 1000 | 500 | 250 | 125 |
| AsR117 | 1.42 | 0 | 0 | 0 |
| AsR416 | 1.1 | 0 | 0 | 0 |
| AsR498 | 5.3 | 0 | 0 | 0 |
| PBS | 0 | 0 | 0 | 0 |
| Triton X-100 | 100 | 100 | 100 | 100 |

**Table S3. *Pinellia ternata* antibacterial protein amino acid sequences**

| Protein name | Amino acid sequence | Amino acid number |
|---|---|---|
| PtR1259 | GVVFGLVFAVISESCFSCSGENVSKDLCG | 29 |
| PtR280 | IKLSSCVRACIVLHGMHASISARKHIECRDSETLACLS YVLHVRWPSCDSVRAVLNLYCGLLQFVLAKSTISSEK | 75 |
| PtR325 | ANQNLVKYMQLEFFIQLQ | 18 |
| PtR743 | FGEIILISLLNFIDCVE | 17 |
| PtR594 | NTPFLFHHLIQAYWESMYFMG | 21 |
| PtR840 | LSCLSTDATYYGTLNGISKFIFDYNQSNI | 29 |
| PtR857 | YRLMRWIFVCCRDWGVYHIIFVDISVSEFRLSANLCF FSKERWLATFSQKTFANSKVLFFPINGSTSYYLS | 71 |
| PtR1776 | MLGGENIITVDGHISYS | 17 |

**Table S4. Primers for *A. sativum* and *P. ternata* library construction using the *B. subtilis* expression system**

| | |
|---|---|
| Oligo dT（contains *Xba* I enzyme site） | ACAGGCTCTAGAGCTTTTTTTTTTTTTTTTTTTTTTT |
| Adapter 1 (contains *Nde* I cleavage site) | CTCGAGAGGAATTCCATATGC |
| Adapter 2 (contains *Nde* I cleavage site) | GCATATGGAATTCCTCTCGAGTACG |
| Adapter 3 (contains *Nde* I cleavage site) | CTCGAGAGGAATTCCATATGCT |
| Adapter 4 (contains *Nde* I cleavage site) | AGCATATGGAATTCCTCTCGAGTACG |
| Adapter 5 (contains Nde I cleavage site) | CTCGAGAGGAATTCCATATGCTA |
| Adapter 6 (contains *Nde* I cleavage site) | TAGCATATGGAATTCCTCTCGAGTACG |

**Table S5. Primers for *A. sativum* library construction using the *E. coli* expression system**

| | |
|---|---|
| Oligo dT (contains *Not* I enzyme site) | ATAAGAATGCGGCCGCTTTTTTTTTTTTTTTTTTTTTTVN |
| Adapter I (contains *EcoR* I cleavage site) | ATGGAAGGTCCGGAATTCG |
| Adapter2 (contains *EcoR* I cleavage site) | CGAATTCCGGACCTTCCATTTA |
| Adapter3 (contains *EcoR* I cleavage site) | ATGGAAGGTCCGGAATTCGA |
| Adapter 4 (contains *EcoR* I cleavage site) | TCGAATTCCGGACCTTCCATTTA |
| Adapter 5 (contains *EcoR* I cleavage site) | ATGGAAGGTCCGGAATTCGAA |
| Adapter 6 (contains *EcoR* I cleavage site) | TTCGAATTCCGGACCTTCCATTTA |

**Supplementary methods**

**Construction of *B. subtilis* system cDNA libraries**

The leaves of *A. sativum* were infected with *R. solani*, and samples were collected at different time intervals (0 to 72 h at 6 h intervals). Total RNA was extracted by Trizol reagent and after that mRNA was purified by using PolyATtract® mRNA isolation systems (Promega). The cDNA library was created by using PrimeScript™ double strand cDNA synthesis kit (TaKaRa) (Fig. S5) with specific Oligo dT primer (containing *Xba* I cleavage site). Three pairs of adaptor containing *Nde* I cleavage sites were added to cDNA (Table S4). cDNA and vector pBE-S were digested with *Xba* I and *Nde* I restriction endonucleases enzymes. The linear plasmid and cDNA purified using gel recovery kit and clean-up kit. The target fragments were ligated with the vector using $T_4$ DNA ligase. The ligation mixture was first transformed into *E. coli* HST08 competent cells for replication. From positive colonies, plasmids were extracted and transformed to *B. subtilis* WB800 competent cells, cultured overnight on LB plates with kanamycin (10 μg/mL). The recombinant clones were picked from LB plates and shaken in liquid LB medium containing kanamycin, stored in -80 °C refrigerator.

**Construction of *E. coli* system cDNA library**

For the construction of cDNA library by using *E. coli* expression system, the difference from the *B. subtilis* secretory protein expression system is that the cDNA and the vector pET22-(b) were cleaved with *EcoR* I and *Not* I restriction endonucleases (Table S5). After cDNA and vector ligation, the final products were first transformed into HST08 competent cells and then transformed into *E. coli* DE3 competent cells. The plasmid was extracted and stored at -20 °C.

**Screening of *E. coli* system cDNA library**

After the plasmid was transformed into DE3, the bacterial cells were diluted $10^3$ fold. Then, 100 μl of bacteria was taken and inoculated onto a membrane (0.45

µm pore size) fixed on LB plates containing appropriate amount of ampicillin. The plates were incubated at 37 °C for 14 h until small colonies (0.2 - 0.4 mm) appear on the membrane. Later on, membrane was transferred to the LB plates containing appropriate amount of ampicillin and 1 mM IPTG. The plates were again incubated at 37 °C for 3 - 4 h, until the colonies grow to normal size. Finally, membranes were transferred to staining agar medium [0.4 % (w/v) agar, 0.025 % (w/v) bromophenol blue and trypan blue], stained for 10 minutes, carefully observed and blue colonies were collected in LB broth containing appropriate amount of ampicillin.

**Protein extraction of *B. subtilis* expression system**

A loop of strain from -80 °C stock was streaked on the LB plate containing kanamycin. Plate was incubated at 37 °C for 16 h. Colonies on the plate were collected into 50 ml LB medium containing kanamycin, and incubated at rotary shaker at 37 °C for 8 - 12 h. Then 2 ml culture was taken into 200 ml LB medium containing kanamycin and again incubated at rotary shaker at 37 °C for 72 h. After 72 h, 15 ml of culture was collected in centrifuge cups and centrifuged at 10,000 rpm, 4 °C for 25 minutes. Supernatant was collected into a precool beaker on ice and saturated ammonium sulfate was added with continuous stirring until the solution becomes turbid. Then, beaker was placed at 4 °C refrigerator overnight to precipitate the protein. The whole solution in the beaker was collected in 50 ml centrifuge cup and centrifuged at 4 °C, 10,000 rpm for 25 minutes. The supernatant was removed and the precipitate was dissolved with 1 ml precooled PBS buffer. Protein (including control proteins) concentration was determined with a spectrophotometer and adjusted with PBS solution. The extracted protein was stored at 4 °C for further use.

**Protein extraction of *E. coli* expression system**

Transformed *E. coli* cells were growth on the membrane over LB plates containing ampicillin. Then, plates were incubated at 37 °C for 24 h. After 24 h,

membrane was transferred to fresh LB plates containing ampicillin with and without IPTG (as control). After 3 h of culture, staining was performed with staining agar medium. If the plate is full of blue single colonies that proved the gene is induced and expressed. Then colonies on the LB/AMP (control) and LB/AMP/IPTG plates were scraped off with sterile blades and collected into two 1.5 ml tubes, respectively. Volume of each culture was recorded and stored at -20 °C. The frozen samples were thawed on ice and equal volume of lysate (20 mM HEPES, pH 7.6, 500 mM NaCl, 10 % glycerol, 1 mM EDTA, 1 Mm PMSF, 5 µg/ml leupeptine, 1 % v/v Aprotinin, and 0.1 % NP40) as the sample volume was added. After vortexing and stirring, the sample was put into liquid nitrogen for several seconds and thawed in 4 °C water. Above procedure was repeated 6 times to completely break the cells. After centrifugation for 10 minutes, 4 °C and 12000 rpm, and then supernatant was collected into 1.5 ml tubes and stored at 4 °C.

**Antibacterial bioassay**

Indicator bacteria were shaken for 8 - 10 h and concentrations were adjusted with spectrophotometer. The NA plates were prepared and 300 µl of indicator bacteria mixed with 4 ml of semi-solid NA medium poured into upper layer of the NA plates. All protein samples were filtered with a 0.22 µm bacterial filter and 20 mg aliquots of protein were dropped onto the filter paper (0.5 cm in diameter). Antibacterial activity was detected after incubated at 37 °C for 12 h and inhibition zones were measured. For the heated-protein antibacterial bioassay, extracted protein was heated at different temperatures, 4 °C, 30 °C, 50 °C, 70 °C and 100 °C in water bath for 15 minutes and, then spotted on the plates inoculated with *B. subtilis* 168. For antifungal bioassay, procedure was similar with antibacterial bioassay. Fungi were inoculated onto the medium with an inoculation loop. Added 50 µl protein until fungi grow to about 1 cm in diameter.

## SEM

Target gene *AsR*416 causing autolysis and control *As*118 were cultured at 37 °C for 36 h. While *AsR*E67 induced with and without IPTG, were cultured in LB medium at 16 °C for overnight. Both cells harvested by centrifugation at 5000 rpm for 3 minutes. Bacterial cells were fixed for 2 h at room temperature in 2.5 % v/v glutaraldehyde in 0.1 M sodium phosphate buffer (pH 7.2). After washing 3 times with 0.1 M, 0.05 M and 0.025 M of phosphate buffers for 15 minutes each, the fixed samples were dehydrated through a graded ethanol series of 30, 50, 70, 90 and 100 % v/v for 15 minutes each and then samples were kept in desiccators overnight. Dehydrated samples were observed by JEOL JSM-7001F Scanning Electron Microscope after coated with gold.

## Percent haemolytic analysis

Briefly, haemolytic activities were tested with freshly prepared sheep red blood cells. Sheep red blood cells were collected then washed three times with PBS (35 mM phosphate buffer, 150 mM NaCl, pH 7.4) and diluted up to a final concentration of 1 %. Then, 70 μl of erythrocytes suspensions and 70 μl of serial dilutions of proteins (1000，500，250，125，62.5 μM) were added to each well of a 96-well plate, and incubated at 37 °C for 1.5 h. The results were observed at 540 nm by microplate reader.

## Western blot

The isolated proteins were electroblotted onto PVDF membranes and the protein transfer was confirmed by using a color pre-stain marker. PVDF membranes were blocked in 2.5 % nonfat dry milk for 2.5 h, transfer to nonfat dry milk mouse containing mouse primary antibody overnight, washed three times in phosphate-buffered saline with 0.1 % Tween-20 (PBST) for 10 - 15 minutes. After incubation with peroxidase-conjugated goat anti-mouse antibody for 3 h followed by three washes with PBST and detection by enhanced

chemiluminescence (ECL) western blot assay. Gel imager was used to detect the bands.
